# A novel extreme adaptive GRU for multivariate time series forecasting

**DOI:** 10.1038/s41598-024-53460-y

**Published:** 2024-02-05

**Authors:** Yifan Zhang, Rui Wu, Sergiu M. Dascalu, Frederick C. Harris

**Affiliations:** 1 Department of Computer Science and Engineering, University of Nevada, Reno, NV 89557 USA; 2 Department of Computer Science, East Carolina University, Greenville, NC 27858 USA

**Keywords:** Computer science, Information technology

## Abstract

Multivariate time series forecasting is a critical problem in many real-world scenarios. Recent advances in deep learning have significantly enhanced the ability to tackle such problems. However, a primary challenge in time series forecasting comes from the imbalanced time series data that include extreme events. Despite being a small fraction of the data instances, extreme events can have a negative impact on forecasting as they deviate from the majority. However, many recent time series forecasting methods neglect this issue, leading to suboptimal performance. To address these challenges, we introduce a novel model, the Extreme Event Adaptive Gated Recurrent Unit (eGRU), tailored explicitly for forecasting tasks. The eGRU is designed to effectively learn both normal and extreme event patterns within time series data. Furthermore, we introduce a time series data segmentation technique that divides the input sequence into segments, each comprising multiple time steps. This segmentation empowers the eGRU to capture data patterns at different time step resolutions while simultaneously reducing the overall input length. We conducted comprehensive experiments on four real-world benchmark datasets to evaluate the eGRU’s performance. Our results showcase its superiority over vanilla RNNs, LSTMs, GRUs, and other state-of-the-art RNN variants in multivariate time series forecasting. Additionally, we conducted ablation studies to demonstrate the consistently superior performance of eGRU in generating accurate forecasts while incorporating a diverse range of labeling results.

## Introduction

The forecasting of time-series data holds immense significance in contemporary society, with research findings in this domain offering versatile solutions to practical challenges. For instance, the development of real-time wildfire spread forecasting systems is indispensable for proactive risk management^1^, while nitrate prediction models play a pivotal role in efficient water resource management^2^. Accurate time series forecasting models assume a vital role in safeguarding lives and property. In recent years, deep learning has showcased its superior capabilities compared to conventional methods in the domain of time series forecasting^3^. Specifically, Recurrent Neural Networks (RNNs), designed for processing sequential data, have emerged as a competitive approach for tackling time series forecasting problems. Rumelhart et al.^4^ introduced the vanilla RNN, which shares the same weights at all time steps and then updates the weights using the back-propagation algorithm. Long short-term memory (LSTM) is a commonly used type of RNN. For example, Zhao et al.^5^ applied LSTM to short-term traffic forecasting to learn temporal-spatial correlation in traffic systems. Cho et al.^6^ proposed a Gated Recurrent Unit (GRU), which builds on LSTM, has a simpler architecture, and is more efficient. Our proposed eGRU is based on GRU and the main goal is to assist RNNs to differentiate between normal and extreme events.

Extreme events, such as hurricanes, system failures, and stock market crashes, are infrequent yet profoundly impactful events that have considerable influence on data patterns. Machine learning techniques have been proposed to leverage historical data patterns for various applications^7,8^. Nonetheless, it is imperative to recognize that data patterns can significantly diverge between normal and extreme events. Consequently, an effective time series forecasting method should possess the capability to differentiate and predict extreme events within the data stream. Despite numerous studies dedicated to detecting normal and extreme events, a comprehensive literature review^9^ highlights the limited research addressing the customization of machine learning models to efficiently capture the distinctions between extreme and normal events for forecasting tasks. For time series forecasting problems, researchers often overlook the fact that extreme events can mislead a machine learning model because their distribution is different from the vast majority of data instances. Consequently, most methods proposed in recent years analyze extreme and normal events indiscriminately. An alternative strategy often employed involves the exclusion of all extreme events from the dataset. However, this practice comes with a drawback-it prevents the model trained with extreme events^10^. Ding et al.^11^ described the heavy-tailed distributions, and concluded that the previous deep learning methods neglected the extreme events and failed to model the tail. They also pointed out that insufficient data for extreme events is the reason that the performance of deep learning time series forecasting methods is degraded. Zhang et al.^12^ introduced a framework that utilizes two distinct machine learning models for the prediction of normal and extreme events in time series data. Given the scarcity of extreme events, the machine learning model trained on limited extreme events data suffers from underfitting.

In this paper, we propose a novel extreme event adaptive gated recurrent unit called eGRU for multivariate time series data forecasting. The eGRU architecture is derived from the vanilla GRU but is customized to maintain two distinct hidden states for modeling normal and extreme events. This enables the eGRU to effectively capture the distinct data patterns of normal and extreme events while preserving temporal information within the input sequence.

The main contributions of this paper are as follows:

- We propose a novel GRU architecture, denoted as eGRU, specifically designed for time series forecasting tasks. The proposed eGRU models normal and extreme events separately while preserving the inherent temporal information of the time series data. This is achieved by utilizing a shared set of weights and biases and maintaining two separate hidden states to capture the dynamics of normal and extreme events independently.
- We introduce a time series data segmentation technique that partitions an input sequence into segments. the eGRU aims to learn the pattern of segments, which consist of multiple time steps, rather than focusing on individual time steps.
- We performed extensive experiments to evaluate the effectiveness and robustness of our proposed eGRU architecture in multivariate time series forecasting. The experimental results demonstrate that eGRU can leverage temporal information within normal and extreme events, leading to superior performance compared to RNN, LSTM, GRU, and state-of-the-art RNN variants. Furthermore, the ablation study results demonstrate that the accuracy of extreme event labeling has minimal impact on the time series forecasting accuracy of the eGRU, thereby indicating its robustness in this regard.

## Methods

This section provides a detailed introduction to the proposed eGRU method for time series forecasting. We begin by introducing the one-layer eGRU framework and discuss the time series segmentation operation. Next, we then explain the architecture of the eGRU cell. Finally, we detail the forward and backward propagation processes of the eGRU layer and discuss how it benefits time series forecasting tasks.

### eGRU framework

The multivariate time series (MTS) forecasting task aims to generate predictions ${\textbf{X}}_{pred} \in {\mathbb {R}}^{O \times D}$ for future *O* time steps by modeling historical records $X \in {\mathbb {R}}^{I \times D}$.

**Figure 1:**
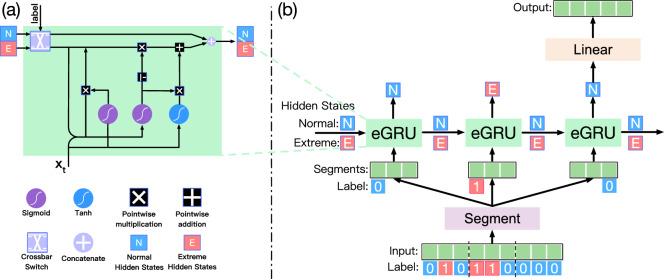
(**a**) The architecture of the eGRU cell. (**b**) Illustration of the proposed eGRU framework.

In Fig. 1b, we present a framework comprising a one-layer eGRU, with each eGRU step representing an eGRU cell, and its detailed architecture is illustrated in Fig. 1a. The input to the framework, denoted as $X \in {\mathbb {R}}^{I \times D}$, represents an MTS sequence of length *I* and comprises *D* variables. The binary label input for the eGRU, denoted as $L \in {\mathbb {R}}^{I}$, with values of either 0 or 1, representing normal and extreme time steps, respectively. The detailed label generation algorithm for time series data utilizing percentile thresholds is introduced in the Supplementary (Section 1). The eGRU framework initiates with the time series segmentation step, wherein the input sequence is partitioned into segments containing values at multiple time steps.

#### Time series segmentation

For RNN-based time series forecasting tasks, the conventional approach involves using a single time step iteration of the RNN. However, it’s important to note that time series data inherently carry less semantic information compared to other data types, such as images or words. Analyzing time series data typically involves examining patterns over a period, such as how variables change within a specific time window and the characteristics they exhibit during that time. This inherent nature of time series data makes it challenging to effectively capture data patterns when modeling the hidden state at an individual time step resolution. Another drawback of analyzing time series data at a single time step resolution is that the length of the input sequence may be very long, for example, the data sampled 6 times per hour has a total of 1,008 time steps in one week. The RNN model is naturally susceptible to gradient vanishing and gradient explosion as the length of the input sequence increases. This leads to increased complexity in the training process and makes convergence more challenging.

To address these issues, we introduce time series data segmentation for RNN which can also be used for our proposed eGRU framework shown in Fig. 1b. Given an input time series sequence *X* and label *L* of length *I*, and a segment size of *p*, the segment operation partitions the entire input sequence into segments as follows:

1 $$\begin{aligned} \begin{aligned} {\textbf{X}}_{seg}&={\text {Segment}}\left( {\textbf{X}}, {\textbf{X}}_{0}, p\right) \\ {\textbf{L}}_{seg}&={\text {Mode}}\left( {\text {Segment}}\left( \mathbf {L, {\textbf{L}}_{0}}, p\right) \right) \end{aligned} \end{aligned}$$

Here, ${\textbf{X}}_{seg} \in {\mathbb {R}}^{T \times p \times D}$ represents MTS segments, with $T = \lceil I/p \rceil$ being the number of segments. ${X}_{0}$ denotes zero-padding, applied in case the sequence length *I* is not divisible by the segment size *p*. The label *L* undergoes an additional ${\text {Mode}}$ procedure to generate segments’ label ${\textbf{L}}_{seg} \in {\mathbb {R}}^{T}$. For example, the input sequence length in Fig. 1b has 9 time steps, it is divided into 3 segments, each with a length of 3. The label for segments is determined as the mode value among the labels of individual segments.

#### Extreme adaptive sub-series temporal dependencies

As a consequence of the ${\text {Segment}}$ procedure, our proposed eGRU operates in a channel-independent manner, in contrast to existing RNN-based methods. While traditional approaches consider values of multiple variables at a single time step as input for each iteration, the eGRU takes values of a single variable across multiple time steps as input for each iteration. Consequently, the input length is dramatically reduced to just 1/*p* of the original sequence. This reduction significantly alleviates the issue of gradient explosion commonly associated with the eGRU. This distinctive feature allows the eGRU to operate independently for each variable, with a specific focus on modeling the temporal data patterns for individual variables. The eGRU iteratively processes time series segments of individual variables, producing output hidden states as follows:

2 $$\begin{aligned} {\textbf{h}}_d={\text {eGRU}}_d\left( {\textbf{X}}^d_{seg}, {\textbf{L}}_{seg}\right) \end{aligned}$$

Where ${\textbf{X}}^d_{seg}$ is the *d*-th series in the MTS data, ${\text {eGRU}}_d$ is the *d*-th eGRU model designed to capture temporal dependencies in the univariate series, and ${\textbf{h}}_d$ is the output hidden state. Note that eGRU has two hidden states for normal and extreme events, respectively. The output hidden state is selected according to the label of the input. The hidden state from the last iteration is chosen to generate predictions for the target time step. The eGRU framework does not require a label for the prediction target. For example, the single-layer eGRU illustrated in Fig. 1b receives three labeled segments as input. Each eGRU step processes a segment and its associated label, subsequently updating and outputting the corresponding hidden state (as shown in Fig. 1a). The eGRU layer outputs normal hidden states in the last step of Fig. 1b, which are subsequently employed to generate time series forecasts.

#### Forecast inference

Finally, a linear layer is employed to establish the mapping between the hidden state and the output forecasts as follows:

3 $$\begin{aligned} \begin{aligned} {\textbf{X}}^{d}_{pred}&={\text {Linear}}\left( {\textbf{h}}_d\right) \\ {\textbf{X}}_{pred}&={\text {Concat}}\left( {\textbf{X}}^{1}, \ldots , {\textbf{X}}^{d}, \ldots , {\textbf{X}}^{D}\right) \end{aligned} \end{aligned}$$

Here, ${\textbf{X}}^{d}_{pred} \in {\mathbb {R}}^{O \times 1}$ denotes the predictions for the *d*-th variable over a forecast horizon of *O* time steps. The overall prediction ${\textbf{X}}_{pred} \in {\mathbb {R}}^{O \times D}$ is obtained by concatenating predictions for all *D* variables.

### eGRU cell architecture

The eGRU architecture maintains two distinct hidden states, referred to as normal and extreme states, with the specific purpose of modeling normal and extreme events within the input time series data. In an RNN, the hidden state functions as a latent representation that undergoes updates at each iteration based on the input. The vanilla GRU architecture models all the time steps with a single hidden state *h*, making it ineffective in capturing the distinct data patterns associated with normal and extreme events. In contrast to the vanilla GRU architecture, our proposed eGRU employs distinct hidden states for extreme events ($h^E$) and normal events ($h^N$). This design empowers the eGRU to model the normal and extreme events independently, thereby enhancing its capacity to accurately capture the characteristics of both types of data instances. It is worth mentioning that eGRU preserves the temporal information within the input sequence, resulting in an accurate representation of the data through its hidden states. The accurate representation of the data through the proposed eGRU’s hidden states advances the accuracy of time series forecasting.

In Fig. 1a, the architecture of the eGRU cell is illustrated. It requires two inputs: (1) a length *p* time series segment, denoted $x_t$, and (2) the corresponding label of the segment, $l_t$. The first procedure of eGRU is to determine the label of the current iteration. It has a label $l_t$ indicating whether the input is a normal or an extreme event. We denote the hidden state from the previous iteration $t-1$ as $h^N_{t-1}$ and $h^E_{t-1}$. Then, the corresponding hidden state for the eGRU cell is chosen as follows:

4 $$\begin{aligned} h_{t-1} = {\left\{ \begin{array}{ll} h^E_{t-1}, &{} \text {if }l_t = 1 \\ h^N_{t-1}, &{} \text {if }l_t = 0 \end{array}\right. } \end{aligned}$$

where $h_{(t-1)}$ is the hidden state to be updated. This procedure is performed by a $2 * 2$ crossbar switch. It takes two signals and a label value as inputs. The crossbar switch will exchange the path of two signals if the label value is 0. This operation enables the eGRU cell to update the hidden state based on the label at iteration *t*. Once the hidden state is determined, the computation within the eGRU cell remains consistent with that of the vanilla GRU cell, as outlined below:

5 $$\begin{aligned} \begin{aligned} r_t&= \sigma (W_{ir}x_t + B_{ir} +W_{hr}h_{t-1} +b_{hr}) \\ z_t&= \sigma (W_{iz}x_t + B_{iz} +W_{hz}h_{t-1} +b_{hz})\\ n_t&= tanh(W_{in}x_t +b_{in} +r_t*(W_{hn}h_{t-1} + b_{hn})) \\ h_t&= (1 - z_t)*n_t + z_t*h_{t-1} \end{aligned} \end{aligned}$$

where $r_t$, $z_t$, and $n_t$ are intermediate variables that represent reset, update, and new gates, respectively. $h_t$ is the hidden state at iteration *t* obtained from input $x_t$, and hidden state $h_{t-1}$ is at iteration $t-1$. $\sigma$ and *tanh* are logistic sigmoid and hyperbolic tangent activation functions, respectively. Lastly, $*$ is the element-wise product. The eGRU cell contains two sets of weights and biases, $W_{ir}, B_{ir}, W_{iz}, B_{iz}, W_{in}, b_{in}$ are the parameters for the input sequence, and $W_{hr}, b_{hr}, W_{hz}, b_{hz}, W_{hn}, b_{hn}$ are for the hidden state. After the hidden state $h_t$ is obtained, the eGRU then updates the normal and extreme hidden states as follows

6 $$\begin{aligned} \begin{aligned} h^E_t, h^N_t&= {\left\{ \begin{array}{ll} h_t, h^N_{t-1} &{} \text {if }l_t = 1 \\ h^E_{t-1}, h_t &{} \text {if }l_t = 0 \end{array}\right. } \\ \end{aligned} \end{aligned}$$

where $h^N_t$ and $h^E_t$ are normal and extreme hidden states, respectively.

### eGRU layer architecture

The eGRU layer requires two inputs: (1) the multivariate time series data $x_{t-T:t}$ where *T* is the input sequence length, and (2) the label $l_{t-T:t}$. For each iteration of the eGRU from $t-T$ to *t*, the calculation follows Eqs. (4)–6. At one iteration, either the normal or extreme is updated based on the corresponding label. By updating normal or extreme hidden states step-by-step, we obtained both normal and hidden states for the whole input sequence.

**Figure 2:**
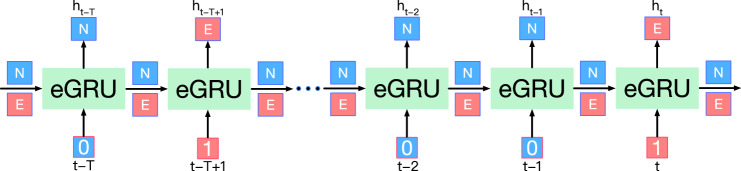
Illustration of eGRU layer.

Figure 2 is an example to show the workflow of a single layer eGRU with the input sequence $t-T, t-T+1, \ldots , t-2, t-1, t$. The input sequence starts at step $t-T$, and the eGRU loads the initial normal and extreme hidden states $h^N_0$ and $h^E_0$. Since the label $l_{t-T}$ is 0, the normal hidden state $h^N_{t-T}$ is updated. On the other hand, the extreme hidden state $h^E_{t-T}$ remains the same with $h^E_0$. The normal hidden state $h^N_{t-T}$ is the output $h_{t-T}$ at iteration $t-T$. Next, iteration $t-T+1$ is an extreme event. Therefore, the eGRU loads $h^E_{t-T}$ from previous iteration and updates $h^E_{t-T+1}$. The output $h_{t-T+1}$ is the extreme hidden state. The subsequent iterations follow the same procedure until it reaches the last iteration *t*. After working through the whole input sequence, an eGRU layer obtains the layer output and the hidden states of the final iteration. In the example shown in Fig. 2, layer outputs are $\{h^N_{t-T}, h^E_{t-T+1}, \ldots , h^N_{t-2}, h^N_{t-1}, h^E_t\}$ containing the hidden states of the whole input sequence.

From an eGRU layer, we obtained normal and extreme hidden states that are aimed at modeling the normal and extreme events, respectively. Note that we used the same set of parameters to update normal and extreme events. From the perspective of the input sequence, the eGRU updates its weights and biases at each time step. Therefore, eGRU can also learn the temporal pattern in the input sequence like a vanilla GRU.

### The backpropagation mechanism of eGRU

For RNN, the back-propagation of multivariate time series can be denoted as follows.

7 $$\begin{aligned} \frac{\partial h^{(t)}}{\partial h^{t-T}} = W^{T-1} \end{aligned}$$

It is susceptible to gradient exploding or vanishing when the largest eigenvalue of *W* is greater or smaller than 1, respectively. Although vanilla GRU mitigates this issue to some extent, it still necessitates gradient back-propagation through every iteration in the input sequence, making it vulnerable to this problem when the input sequence length becomes substantial. In contrast, eGRU updates either the normal or extreme hidden states within a single iteration. In other words, the hidden state can be viewed as skipping several iterations. Therefore, when the gradient back-propagates from output to input, the iterations are less than the total input length *T*. The occurrence of extreme events is significantly less than normal events, e.g. 10%. The gradients for extreme events similarly only need to go through 10% of all the steps.

**Figure 3:**
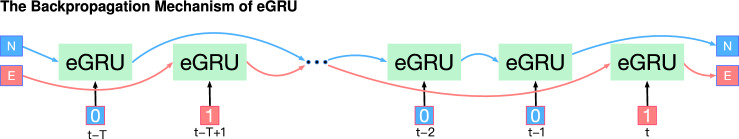
The eGRU back-propagation mechanics illustration.

For example, the step at $t-2$ in Fig. 3, its gradient $h^N_{t-2}$ is calculated as follows.

8 $$\begin{aligned} \frac{\partial h^N_t}{\partial h^N_{t-2}} = \frac{\partial h^N_t}{\partial h^N_{t-1}}\frac{\partial h^N_{t-1}}{\partial h^N_{t-2}} \end{aligned}$$

where $h^N_t$, $h^N_{t-1}$, and $h^N_{t-2}$ are the normal hidden state for step *t*, $t-1$, and $t-2$, respectively. The label of step *t* in Fig. 3 is extreme. Therefore, $h^N_t$ and $h^N_{t-1}$ are identical, the $t-1$ step in Eq. (8) can be omitted, the gradient backpropagation directly calculated from hidden states at step *t* to $t-2$.

As shown in Fig. 3, eGRU outputs only a normal or extreme hidden state at one iteration in forward propagation. Therefore, in backward propagation, the gradient is only backward propagated from the blue (i.e., normal) or red line (i.e., extreme). That is, for which output is the extreme hidden state, all normal steps are skipped in the back-propagation. Similarly, for which output is the normal hidden state, all the extreme steps are skipped. By the skip connection, the total orders of Eq. (7) are reduced for normal and extreme events. The total number of steps that both the extreme and normal gradients traverse is equal to the length of the input.

## Results

In this section, we present experimental results on four real-world multivariate time-series datasets to demonstrate the effectiveness of our proposed eGRU for multivariate time-series forecasting. Our experiments indicate that eGRU outperformed state-of-the-art RNN variants in terms of forecasting accuracy. Furthermore, we conducted an extensive ablation study to evaluate the effectiveness of the main components of eGRU and important hyperparameters.

### Datasets and metrics

We utilized the following four publicly available benchmark datasets^13^ with different characteristics to evaluate the performance of the proposed eGRU.

*Solar-energy:* This dataset collects the production data points for 137 solar power plants in the state of Alabama, with a sample rate of 10 minutes, for the year 2006. A total of 52,560 samples. An intriguing feature of this dataset is the occurrence of zero values in the evening.

*Traffic:* This dataset contains road occupancy rate measurements (between 0 and 1) of 862 freeway sites in the San Francisco Bay area. Hence, the 862 variables in the datasets exhibit spatial correlation. The California Department of Transportation provides over ten years of publicly available data. Lai^13^ collected hourly data for 48 months (2015-2016), yielding 17,544 samples in total.

*Electricity:* UCI Machine Learning Repository provides this dataset which contains the electricity consumption of 321 clients measured in kWh every 1 hour from 2012 to 2014. The electricity consumption exhibits a distinct daily pattern. A total of 26,304 samples.

*Exchange-rate:* This dataset reflects for 27 years (1990-2016) of daily exchange rates of 8 major world economies: Australia, Britain, Canada, Switzerland, China, Japan, New Zealand, and Singapore. This dataset contains 7,588 samples in total. This dataset is unique in its absence of discernible seasonal patterns. Conversely, it demonstrates a pronounced degree of locality.

The datasets were partitioned into distinct subsets, namely the training set, validation set, and test set, with a ratio of 0.6, 0.2, and 0.2, respectively. The training set was employed for model training, and the optimal model was selected based on the minimum loss function value observed on the validation set. Subsequently, the saved optimal model was loaded for testing on the test set, generating a comprehensive set of evaluation metrics that elucidate the model’s performance on the test data.

We utilized three widely adopted metrics^14^ to evaluate time series forecasting tasks: relative squared error (RSE), relative absolute error (RAE), and empirical correlation coefficient (CORR). These metrics were employed to evaluate the performance of both the proposed eGRU model and other baseline models. Lower values of RAE and RSE indicate superior performance, while higher values of CORR suggest better model performance.

### Implementation details

The experiments were conducted on a Linux server equipped with a single NVIDIA GeForce RTX 3090 24 GB GPU. The hyperparameter settings used in this study were chosen manually during the experimental procedure. The initial learning rate for all four datasets was set to $1e-3$. The number of hidden units in the RNN was set to 100. The loss function used for the Exchange-Rate dataset was L1 loss, while for the other datasets, L2 loss was utilized. The batch size for all datasets was set to 32, and the input sequence length was set to $24*7$. The segment size for time series segmentation was set to 24. To enable forecasting using the eGRU model, we employ a threshold *k* of $90\%$. The window size *w* and slide steps *s* were set to 1000 and 100, respectively, for the Exchange-Rate dataset, whereas for all other datasets, we set these parameters to 10, 000 and 1000, respectively. In ablation studies assessing the effectiveness of the components and hyperparameters, all settings remained consistent with those previously introduced, except for the specific component or hyperparameter under evaluation.

### Baseline methods

To evaluate the performance of the proposed eGRU, we conducted experiments comparing it with the vanilla RNN, LSTM, GRU, 6 variants of RNNs, and 2 RNN-based frameworks:

- **RNN**^4^: The vanilla RNN architecture iteratively processes input sequences, updates its weights and biases, and generates a hidden state to model the underlying data patterns.
- **LSTM**^5^: The vanilla LSTM architecture introduces the input, forget, cell, and output gates into the vanilla RNN. LSTM employs these four elements to model both long-term and short-term data patterns within the input sequence, saving information as the hidden state and cell state.
- **GRU**^6^: The vanilla GRU architecture employs two gated mechanisms, namely Reset and Update, to sustain a single hidden state that captures the temporal dynamics of the input time series data. By reducing one gated mechanism compared to the LSTM architecture, the GRU architecture exhibits greater efficiency, as it has fewer learnable parameters.
- **SkipGRU**^15^: A novel RNN modified from existing RNN architecture to selectively skip the hidden state updates in the sequential computations. Hence, it shortens the length of the computational sequence.
- **ZoneoutLSTM**^16^: The zoneout technique was applied to the LSTM to stochastic determine whether the hidden state and cell state at a time step should be updated or retained from the previous time step.
- **PhasedLSTM **^17^: The PhasedLSTM is an extension of the vanilla LSTM architecture that introduces a new time gate controlled by a parameterized oscillation. This gate enables updates of the memory cell to occur only during a small portion of the oscillation cycle.
- **IndRNN **^18^: The cells within the same layer are independent and they are connected with cells of other layers.
- **mLSTM **^19^: The mLSTM model integrates a memory filter component that dynamically assigns weights to the time steps of an input sequence.
- **HSNLSTM **^20^: This framework incorporates an LSTM with an adaptive and hybrid spiking module that utilizes spiking neural networks. The embedded LSTM collaborates with two attention mechanisms to enhance its capabilities.
- **LSTNet **^13^: LSTNet is a deep learning framework that leverages CNNs to capture local dependencies among variables and RNNs to learn long-term temporal dependencies. The framework also incorporates a skip RNN architecture, which is designed to capture dependencies between long time intervals. To generate final predictions, LSTNet employs elementwise sum and linear bypass operations.
- **TPA-LSTM **^14^: TPA-LSTM utilizes a single-layer RNN to learn a set of hidden states and subsequently employs a set of CNN filters to extract short-term patterns. The CNN and RNN outputs are integrated using an attention mechanism, allowing the model to learn the weighting and combination of these hidden states, to generate forecasts.

### Main results

Table 1 presents the main experimental results of all methods for four datasets in terms of three metrics. In time series forecasting tasks, predicting time steps that are further into the future is generally more challenging. For this study, we selected forecast horizons of 3, 6, 12, and 24 time steps to assess the performance of methods under varying levels of task difficulty. The best results of each case (dataset, horizon, and metric) are highlighted in bold within the table.

**Table 1:** Comparison results (RSE, RAE, and CORR) of eGRU and baseline methods on four multivariate time series data: (1) Each row represents the results of a method in terms of a specific metric. (2) Each column represents the results of a specific horizon for each dataset. (3) The best results in the table are shown in bold. .

Method	Dataset	Solar-energy				Traffic				Electricity				Exchange-rate			
	Horizon	3	6	12	24	3	6	12	24	3	6	12	24	3	6	12	24
RNN	RSE	**0**.**1988**	0.2744	0.4282	0.7353	0.5642	0.5751	0.5871	0.5981	0.1437	0.1686	0.1476	0.1559	0.0930	0.1227	0.1494	0.1768
	RAE	0.123	0.1758	0.3386	0.7064	0.4432	0.4504	0.4659	0.4892	0.0914	0.1023	0.1019	0.0967	0.0893	0.1159	0.1367	0.1613
	CORR	**0**.**9815**	0.9649	0.9084	0.6808	0.8286	0.8234	0.8095	0.8013	0.8429	0.8146	0.8117	0.8404	0.9652	0.9413	0.927	0.8261
LSTM	RSE	0.204	0.2706	0.3635	0.5394	0.5371	0.5567	0.5696	0.5583	0.1271	0.1348	0.1407	0.1673	0.1291	0.1697	0.1894	0.2079
	RAE	0.1253	0.1766	0.2453	0.4118	0.4041	0.4209	0.4461	0.431	0.0887	0.089	0.0930	0.0955	0.1307	0.166	0.1799	0.1946
	CORR	0.9804	0.9648	0.9341	0.8604	0.8453	0.8374	0.8242	0.8313	0.8289	0.8343	0.8254	0.8325	0.8538	0.8797	0.7184	0.5754
GRU	RSE	0.2007	0.2712	0.3636	0.4997	0.5512	0.5767	0.568	0.5723	0.1236	0.1457	0.1379	0.1559	0.0781	0.1043	0.1247	0.152
	RAE	0.1298	0.1894	0.2734	0.3551	0.4269	0.4597	0.4497	0.4627	0.0888	0.097	0.0959	0.0984	0.0753	0.0980	0.1143	0.1375
	CORR	0.9813	0.9656	0.9349	0.8758	0.8391	0.824	0.8249	0.8219	0.8302	0.8313	0.8124	0.8328	0.9721	0.9558	0.9316	0.8963
SkipGRU	RSE	0.2187	0.2659	0.3813	0.4822	0.5409	0.55	0.5632	0.5665	0.1463	0.172	0.274	0.1552	0.0848	0.1117	0.138	0.1391
	RAE	0.1389	0.1836	0.2745	0.3403	0.417	0.4235	0.4448	0.4574	0.0975	0.109	0.1486	0.0974	0.0806	0.102	0.124	0.1515
	CORR	0.9775	0.9665	0.926	0.8822	0.8427	0.8382	0.8298	0.825	0.8256	0.8151	0.676	0.8349	0.9614	0.9555	0.9301	0.8927
ZLSTM	RSE	0.2525	0.3201	0.4877	0.7661	0.5556	0.6036	0.5821	0.5839	0.1472	0.1764	0.1427	0.1688	0.1437	0.1591	0.1597	0.1751
	RAE	0.1557	0.2334	0.3762	0.7344	0.4154	0.4618	0.4493	0.4508	0.09874	0.1096	0.1015	0.1002	0.1361	0.1516	0.1493	0.1625
	CORR	0.9702	0.9494	0.874	0.6377	0.8381	0.7977	0.8209	0.8129	0.7747	0.7706	0.7844	0.8013	0.8722	0.8166	0.805	0.7216
PLSTM	RSE	0.2048	0.2799	0.3534	0.4944	0.5301	0.5481	0.5544	0.576	0.1476	0.1372	0.1465	0.1531	0.1463	0.1817	0.1949	0.2065
	RAE	0.1236	0.18	0.2381	0.3459	0.3962	0.4107	0.4317	0.4556	0.0905	0.0909	0.0968	0.0922	0.1373	0.1641	0.1764	0.1913
	CORR	0.9803	0.9637	0.9382	0.8681	0.8488	0.8426	0.8342	0.8191	0.823	0.8248	0.8049	0.8263	0.9235	0.814	0.7566	0.5652
IndRNN	RSE	0.2074	0.2829	0.4619	0.6589	0.6445	0.7004	0.6493	0.6388	0.1361	0.1843	0.1715	0.1525	0.0939	0.0986	0.1223	0.1588
	RAE	0.127	0.2018	0.4111	0.6252	0.553	0.6544	0.5502	0.5408	0.09591	0.1177	0.1124	0.0938	0.0876	0.0884	0.1107	0.1438
	CORR	0.9797	0.9624	0.8958	0.7557	0.7883	0.7728	0.7839	0.7817	0.8179	0.8098	0.801	0.8437	0.8651	0.9006	0.8606	0.7551
mLSTM	RSE	0.1997	**0**.**25**	**0**.**3295**	**0**.**4429**	0.5251	0.5726	0.5542	0.5527	0.1214	0.13	0.1327	0.1571	0.1329	0.1657	0.1924	0.2215
	RAE	**0**.**1174**	**0**.**1543**	**0**.**216**	**0**.**3103**	0.389	0.4359	0.4259	0.418	0.0842	0.0869	0.0926	0.0956	0.1278	0.1556	0.1766	0.2071
	CORR	0.9812	**0**.**9697**	**0**.**9454**	**0**.**897**	0.8535	0.8353	0.836	0.8344	0.8278	0.8413	0.8152	0.8121	0.9162	0.8453	0.6581	0.5488
hsnLSTM	RSE	0.2065	0.2514	0.3401	0.4610	0.5526	0.5881	0.5777	0.5841	0.1493	0.141	0.1554	0.1238	0.2543	0.2724	0.2716	0.2608
	RAE	0.1276	0.1657	0.2526	0.3889	0.4539	0.5024	0.4927	0.49	0.0970	0.0964	0.1044	0.0905	0.2313	0.2513	0.2497	0.237
	CORR	0.9797	0.9698	0.943	0.8869	0.8425	0.8206	0.8292	0.8211	0.816	0.819	0.7821	0.7984	0.6624	0.5265	0.4638	0.5553
eGRU	RSE	0.2072	0.2912	0.3922	0.4717	**0**.**4342**	**0**.**4583**	**0**.**4668**	**0**.**4722**	**0**.**0795**	**0**.**0872**	**0**.**0959**	**0**.**1004**	**0**.**0272**	**0**.**0309**	**0**.**0408**	**0**.**0598**
	RAE	0.1215	0.1907	0.2575	0.3292	**0**.**2702**	**0**.**2934**	**0**.**2990**	**0**.**2984**	**0**.**0520**	**0**.**0544**	**0**.**0572**	**0**.**0583**	**0**.**0208**	**0**.**0255**	**0**.**0339**	**0**.**0507**
	CORR	0.9796	0.9570	0.9199	0.8774	**0**.**8929**	**0**.**8802**	**0**.**8752**	**0**.**8725**	**0**.**9166**	**0**.**9008**	**0**.**8848**	**0**.**8836**	**0**.**9792**	**0**.**9707**	**0**.**9560**	**0**.**9338**

For the Solar-Energy dataset, eGRU was better than GRU for horizon 24, while it was worse for horizons 3, 6, and 12. However, mLSTM performed the best for this dataset. We conjecture that this is because the production of solar power plants has a much clearer pattern compared to other datasets, i.e., 0 during nights and peaks during daytime. These clear patterns can be easily learned by other forecasting models, and their performance is similar to or even slightly better than GRU.

For the remaining three datasets, eGRU performed the best for all horizons in terms of all metrics. Taking horizon 24 as an example, Supplementary figure S1 displays the bar plots of RSE, RAE, and CORR for the ten methods. For the Solar-Energy dataset, eGRU outperformed GRU but was outperformed by SkipGRU, mLSTM, and HSNLSTM. For all three datasets, eGRU substantially outperformed GRU and other RNN variants. For instance, when evaluating performance on horizon 24 using Traffic, Electricity, and Exchange-Rate datasets, eGRU outperformed GRU by $17.5\%$, $35.6\%$, and $60.6\%$ in terms of the RSE metric. As for CORR, eGRU improved from GRU by $6.15\%$, $6.1\%$, and $4.2\%$, on these 3 data sets. All variants of RNN performed at the same level as vanilla GRU, while eGRU significantly improved accuracy.

**Figure 4:**
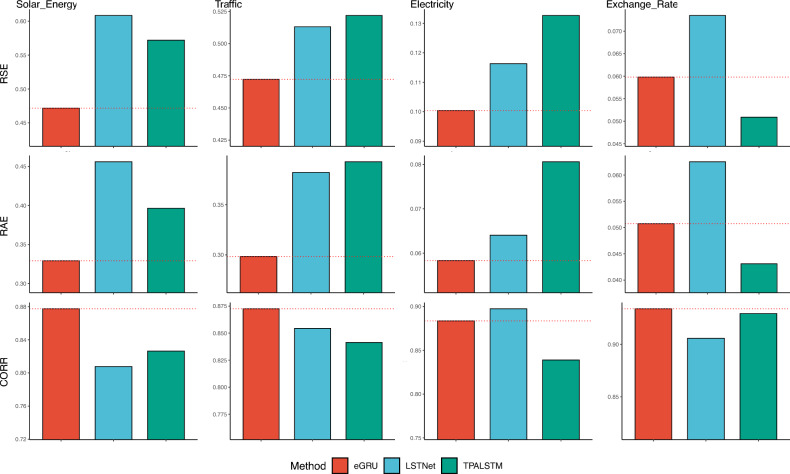
Accuracy assessment of the eGRU, LSTNet, and TPA-LSTM using RSE, RAE, and CORR on horizon 24. The mean value of eGRU is indicated by a dashed red horizontal line.

In addition to RNN variants baselines, we compare the performance of one-layer eGRU with more complex RNN-based models that utilize CNN and attention mechanisms. The performance comparison between eGRU, LSTNet, and TPA-LSTM on horizon 24 is presented in Fig. 4. eGRU outperformed both LSTNet and TPA-LSTM on the Solar-Energy and Traffic dataset in terms of all metrics. For the Electricity dataset, eGRU also achieved better RSE and RAE than both frameworks, only lost to LSTNet in terms of the CORR metric For the Exchange-Rate dataset, eGRU achieved the highest CORR score and outperformed TPA-LSTM in terms of RSE and RAE. Overall, the eGRU model outperformed LSTNet and TPA-LSTM in 9 out of the total 12 cases, indicating its superior performance.

### Ablation study

To demonstrate the effectiveness of the eGRU architecture, we conducted a comprehensive ablation study where we carefully analyzed the main components and essential hyperparameters. This study allows us to gain a deeper understanding of the factors that contribute to the superior performance of eGRU in comparison to other state-of-the-art RNN variants.

*Time series segmentation* An important aspect of the eGRU framework is its time series segmentation design. Supplementary figure S2 shows the performance results of eGRU without the segmentation operation on a horizon of 24. The model, referred to as eGRU-w/oSeq, had the segmentation operation removed. The results indicated that the eGRU without the segmentation operation achieved lower RSE and RAE values for all four datasets and a higher CORR value for the Traffic and Electricity datasets. However, the improvement in performance from the vanilla GRU was overall marginal. On the other hand, the eGRU method presented in Supplementary figure S2, which uses the segment operation, showed significant performance improvement over the vanilla GRU. This demonstrates the effectiveness of the time series segmentation design in the eGRU framework for accurate time series forecasting.

*Extreme events labeling* The labeling of normal and extreme events in time series data presents a challenging task. In the context of time series forecasting, it is not the goal of this paper to compare the quality of extreme event detection results. We assess the forecasting accuracy of eGRU to analyze the impact of extreme event labeling. Experiments were conducted under two scenarios: the first scenario involves applying Algorithm S1 directly to the time series data. In contrast, the second scenario involves applying Algorithm S1 to the anomaly score matrix obtained using the GDN method. The experimental results are presented in Supplementary figure S2.

For eGRU-GDN-w/oSeq and eGRU-GDN, the GDN method was utilized to label normal and extreme events using Algorithm S1. The performance of eGRU-GDN-w/oSeq and eGRU-w/oSeq was similar for the Traffic and Exchange-Rate datasets. While eGRU-w/oSeq performed better than eGRU-GDN-w/oSeq on the Solar-Energy dataset, the opposite was observed for the Electricity dataset. As for the performance of eGRU and eGRU-GDN, it was found that they performed similarly, except for the Exchange-Rate dataset where eGRU outperformed eGRU-GDN significantly.

Even though GDN is a state-of-the-art deep learning-based method that should generate more accurate normal and extreme event labels, our experimental results in the two scenarios are similar. Thus, we conclude that the proposed eGRU architecture is robust to the accuracy of normal and extreme event labeling and consistently produces accurate forecasting results.

*Extreme events threshold* One of the critical parameters for Algorithm S1 is the hyperparameter *k*-th percentile, which determines the threshold for identifying extreme events. A higher threshold results in Algorithm S1 labeling a higher proportion of data instances as normal events and a lower proportion as extreme events. As previously mentioned, detecting extreme events in time series data is a challenging task. Thus, we conducted experiments to evaluate how the threshold affects time series forecasting. Supplementary figure S3 presents the results of various thresholds for three metrics at horizon 24. Overall, we observe that a higher threshold leads to better forecasting accuracy across all three metrics. This observation is consistent with the assumption that extreme events are a small subset of all data instances that occur infrequently and have a different data distribution from the majority.

The thresholds ranging from the 75th to the 90th percentile generally provide the best performance for the proposed eGRU model. Furthermore, the performance of eGRU is highly robust to the choice of thresholds. eGRU incorporates all thresholds that achieved the best performance compared to the baseline methods on the Traffic, Electricity, and Exchange-Rate datasets. For example, on the Electricity dataset, eGRU achieved the highest (worst) RSE and RAE of 0.1036 and 0.0612, respectively, with thresholds of 70 and 55. The best baseline method, HSNLSTM, achieved RSE and RAE of 0.1238 and 0.0905, respectively, which is significantly higher. On the Electricity dataset, the range of RSE, RAE, and CORR is 0.0035, 0.00344, and 0.0076, respectively. Similarly, for the other three datasets, the performance of eGRU varies only slightly with different thresholds ranging from 50 to 90. Thus, we conclude that the eGRU method is highly robust to the choice of thresholds.

Considering the experimental results of ***Extreme events labeling*** and ***Extreme events threshold***, we conclude that the proposed eGRU method exhibits good robustness to the outcomes of extreme event detection. Despite the inherent difficulty of this task, eGRU consistently outperforms baseline methods in most cases.

*Input sequence length* Supplementary figure S4 demonstrates the effect of input sequence length on the accuracy of forecasting. It is important to note that HSNLSTM had some missing results for larger horizons due to its excessive memory utilization. Additionally, we were unable to include mLSTM in this analysis as its code performed significantly slower for longer input sequence lengths.

Generally, eGRU demonstrates more accurate predictions for longer input sequences. However, Supplementary figure S4 reveals distinct trends for the four datasets. The Solar-Energy and Traffic datasets exhibit a sharp decline in accuracy for eGRU when the input sequence length is less than 168 (28 hours for solar and 7 days for traffic). The optimal input sequence length for these datasets is 1176 (8.16 days) and 840 (35 days), respectively. In contrast, the performance of eGRU for the electricity dataset only slightly drops when the input sequence length is shorter than 168. The Exchange-Rate dataset exhibits an intriguing characteristic whereby the accuracy of the eGRU remains relatively constant across varying input sequence lengths. Surprisingly, unlike other datasets, the optimal input sequence length for this dataset is precisely 24. We conjecture that this unique behavior may be attributed to two factors. First, there are no discernible periodic patterns in the Exchange-Rate dataset. Second, the input sequence length of 24 already captures stock price changes for 24 days, given the daily sample rate.

*Size of segmentation* The segmentation of multivariate time series data is a crucial aspect of designing the eGRU. Supplementary figure S5 illustrates the results of an ablation study conducted to evaluate the effects of various sizes of segmentation operations. The effect of segmentation size also exhibits distinct characteristics across different datasets. Specifically, the performance of eGRU on the Solar-Energy dataset demonstrates a steady improvement with increasing segmentation size. The Traffic and Electricity datasets show a similar trend of improved performance with increasing segmentation size until reaching an optimal peak at approximately 42 and 56, respectively, after which performance begins to decline. Notably, on the Exchange-Rate dataset, the performance of eGRU exhibits an opposite trend, decreasing as the segmentation size increases. Notably, we observed that eGRU attained the highest accuracy across all segmentation sizes when applied to the Traffic, Electricity, and Exchange-Rate datasets. This finding highlights the robustness and effectiveness of eGRU in forecasting multivariate time series data.

The segmentation size plays a critical role not only in the accuracy of eGRU but also in its computation time. The iteration of eGRU can be significantly reduced by dividing the input sequence into segments of suitable sizes. For instance, consider an input sequence length of 168 and two different segmentation sizes, 3 and 84. The number of iterations required for segmentation sizes of 3 and 84 would be 56 and 2, respectively. This observation underscores the importance of selecting an appropriate segmentation size to minimize the computational overhead of eGRU while maintaining high accuracy.

We conclude that the performance of eGRU is optimized with longer input sequence lengths and larger segmentation sizes when applied to datasets with periodic patterns. Conversely, for datasets lacking periodic patterns, eGRU’s performance is enhanced with shorter input sequence lengths and smaller segmentation sizes. It is essential to select appropriate input sequence lengths and segmentation sizes based on the characteristics of the dataset to achieve optimal results with eGRU. However, it is worth noting that even though the input sequence length of 168 and segmentation size of 24 are not the optimal selection for any of the datasets as Supplementary figure S5 shows, eGRU still outperformed state-of-the-art RNN variants by a significant margin.

*Robustness against randomness* To assess the robustness of our experimental results against the randomness of eGRU’s weights and biases initialization, we repeated our experiments five times, and the mean and standard deviation values of experimental results in terms of RSE, RAE, and CORR are presented in Supplementary Table S1. Consistent with the main results presented in Table 1, the eGRU model achieved the best average value for all metrics on the Traffic, Electricity, and Exchange-Rate datasets. Moreover, the eGRU model outperformed the other models regarding standard deviation for the Electricity and Exchange-Rate datasets. These results further support the robustness and reliability of the eGRU model for accurate time series forecasting.

## Conclusion

This paper presents a novel RNN architecture, eGRU, for time series forecasting. eGRU outperforms vanilla GRU and other state-of-the-art RNN variants. The proposed architecture leverages two hidden states, normal and extreme, with a shared set of parameters to learn both normal and extreme event data patterns while retaining temporal information. This design enables eGRU to model normal and extreme events independently. Extensive experiments on four real-world datasets, in which eGRU is compared to six state-of-the-art RNN variants and two RNN-based frameworks, demonstrate the superior capacity of eGRU for time series forecasting in terms of accuracy and robustness.

### Supplementary Information

Supplementary Information.

## Data Availability

In this manuscript, we have employed four widely used benchmark multivariate time series datasets that have been prominent in recent state-of-the-art research. Those datasets are readily accessible at https://github.com/laiguokun/multivariate-time-series-data.
