# Supplementary material for: A novel extreme adaptive GRU for multivariate time series forecasting
Source: Sci Rep. 2024 Feb 5;14:2991. doi: 10.1038/s41598-024-53460-y (PMC10844338; doi:10.1038/s41598-024-53460-y)
Supplement: Supplementary file 1 — Supplementary Information. [file 41598_2024_53460_MOESM1_ESM.pdf]

A Novel Extreme Adaptive GRU for Multivariate Time Series Forecasting

Supplementary Material

Yifan Zhang[1,*], Rui Wu[2], Sergiu M. Dascalu[1], and Frederick C. Harris, Jr.[1]

[1] Department of Computer Science and Engineering, University of Nevada, Reno, NV 89557, USA.
[2] Department of Computer Science, East Carolina University, NC 27858, USA.
[*] yfzhang@nevada.unr.edu

Contents

1 Normal and Extreme Events Labeling for Forecasting **3**

2 Extra details of the Main Results **4**

2.1 Formulation of Evaluation Metrics 4

2.2 Visualization of the Main Results 4

3 Visualization of Ablation Studies **6**

References **11**

1 Normal and Extreme Events Labeling for Forecasting

The eGRU architecture requires an additional label vector to determine whether the input segments are normal or extreme, which is essential for its functionality. However, time series data often lacks such labels. Identifying normal and extreme events is essential in forecasting as it allows for accurate prediction of expected events and prepares for unexpected events that may significantly impact the forecast. The definition of extreme events can be subjective; however, from the perspective of time series forecasting, we characterize events that deviate from the majority of data instances as extreme events. We adopt a threshold-based approach, designating events that exceed the threshold as extreme. Specifically, we set the threshold as the 90th percentile of the time series data range.

To identify threshold-based extreme events in a time series data stream, especially when its distribution can evolve over time, we introduce a sliding window detection algorithm, as outlined in Algorithm 1.

Algorithm 1 Labeling normal and extreme events in multivariate time series data using percentage thresholds. All values with percentiles greater than $k\%$ will be marked extreme.

Input: Target matrix $X \in R^{N \times M}$, Window size w, slide step s, Percentage $k\%$.
Output: Label vector: $L \in R^N$

initialize: $K \leftarrow R^{N \times M}$, $L \leftarrow R^N$
for each $j \in \{1, 2, \ldots, M\}$ **do**
    $K_{1:w,j} \leftarrow min(X_{1:w,j}) + (max(X_{1:w,j}) - min(X_{1:w,j})) * k\%$
end for
$t \leftarrow w + s$
while $t \leq N$ **do**
    for each $j \in \{1, 2, \ldots, M\}$ **do**
        $K_{t-s:t,j} \leftarrow min(X_{t-w:t,j}) + (max(X_{t-w:t,j}) - min(X_{t-w:t,j})) * k\%$
    end for
    $t \leftarrow t + s$
end while
for each $t \in \{1, 2, \ldots, N\}$ **do**
    for each $j \in \{1, 2, \ldots, M\}$ **do**
        if $X_{t,j} \geq K_{t,j}$ **then**
            $K_{t,j} \leftarrow 1$
        else
            $K_{t,j} \leftarrow 0$
        end if
    end for
end for
for each $t \in \{1, 2, \ldots, N\}$ **do**
    $l_t \leftarrow MajorityVote(K_{t,1:M})$
end for

Algorithm 1 is employed for labeling normal and extreme events in a time series data stream, requiring four inputs: the data matrix X, window size w, step size s, and percentile value k for threshold calculation. The algorithm begins with a window of w time steps and calculates the threshold for extreme events as the $k\%$-th percentile of the values within the window. As the algorithm progresses, the window slides through the time series stream, and the threshold is updated based on the new window. Values for subsequent time

steps within the window are marked as extreme if they surpass the threshold, otherwise, they are classified as normal. This process continues for the entire data stream. Eventually, the output of Algorithm 1 is a vector $L = l_1, l_2, \cdots, l_{N-1}, l_N$, where each value represents either a normal event (0) or an extreme event (1). Note that when applying Algorithm 1 in a data stream during the testing procedure, its step size s needs to be adjusted based on the data sample rate and prediction frequency to ensure that only historical time steps are utilized. For example, if the model needs to predict hourly data every 12 hours, the step size should be set to a value smaller than 12.

Algorithm 1 can be directly applied to the data to generate labels for extreme events. In addition, we leveraged a Graph Neural Network-Based Anomaly Detection (GDN) approach[1] to generate an anomaly score matrix, which was subsequently subjected to Algorithm 1 to enable extreme event labeling. The Algorithm 1 is also able to employ other univariate anomaly detection methods, such as SPOT[2], by modifying line 3 and line 8 in Algorithm 1.

It is worth mentioning that we conducted ablation studies on Algorithm 1 by substituting the direct percentile threshold of data with GDN. Additionally, we explored how Algorithm 1 influences forecasting accuracy when thresholds are varied within the range of 50 to 95. The results illustrate that the eGRU is robust to variations in extreme event labeling and consistently generates accurate predictions compared to the baseline methods.

2 Extra details of the Main Results

2.1 Formulation of Evaluation Metrics

We employed relative squared error (RSE), relative absolute error (RAE), and empirical correlation coefficient (CORR) as the quantitative evaluation metrics. The formulation of those metrics is as follows:

$$RSE = \frac{\sqrt{\sum_{t=1}^{O}\sum_{d=1}^{D}(y_{t,d} - \hat{y}_{t,d})^2}}{\sqrt{\sum_{t=1}^{O}\sum_{d=1}^{D}(\hat{y}_{t,d} - \overline{y_{1:O,1:D}})^2}} \tag{1}$$

$$RAE = \frac{\sum_{t=1}^{O}\sum_{d=1}^{D}|y_{t,d} - \hat{y}_{t,d}|}{\sum_{t=1}^{O}\sum_{d=1}^{D}|\hat{y}_{t,d} - \overline{y_{1:O,1:D}}|} \tag{2}$$

$$CORR = \frac{1}{D}\sum_{d=1}^{D}\frac{\sum_{t=1}^{O}(y_{t,d} - \overline{y_{1:O,d}})(\hat{y}_{t,d} - \overline{\hat{y}_{1:O,d}})}{\sum_{t=1}^{O}(y_{t,d} - \overline{y_{1:O,d}})^2\sum_{t=1}^{O}(\hat{y}_{t,d} - \overline{\hat{y}_{1:O,d}})^2} \tag{3}$$

where O and D represent the forecasting horizon and the number of variables, respectively. $\hat{y}$ and y denote the predictions and the ground truth of the target, respectively. $\overline{y_{1:O,1:D}}$ represents the mean value of the set of values in y. These metrics are commonly utilized in recent state-of-the-art methods. Therefore, we have adopted them to quantitatively evaluate the performance of our proposed eGRU in comparison with baseline methods.

2.2 Visualization of the Main Results

Figure S1 presents a visualization of the main results for horizon 24, as detailed in Table 1 of the main text. In the case of the Solar-Energy dataset, the proposed eGRU secured the third position in RSE, second in RAE, and fourth in CORR. Conversely, for the remaining three datasets, the eGRU outperformed the compared baseline methods significantly.

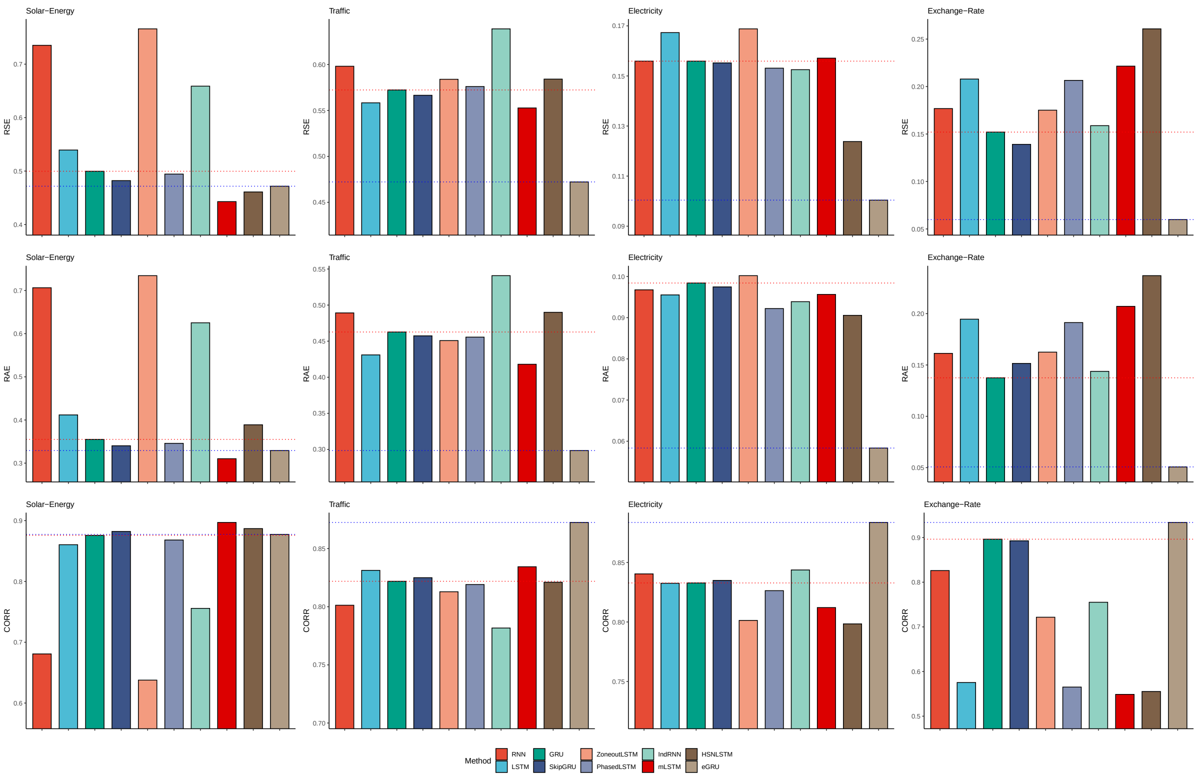

Figure S1. Accuracy assessment of the eGRU and baseline methods using RSE, RAE, and CORR on horizon 24. The mean values of GRU and eGRU are indicated by a dashed red and blue horizontal line, respectively.

3 Visualization of Ablation Studies

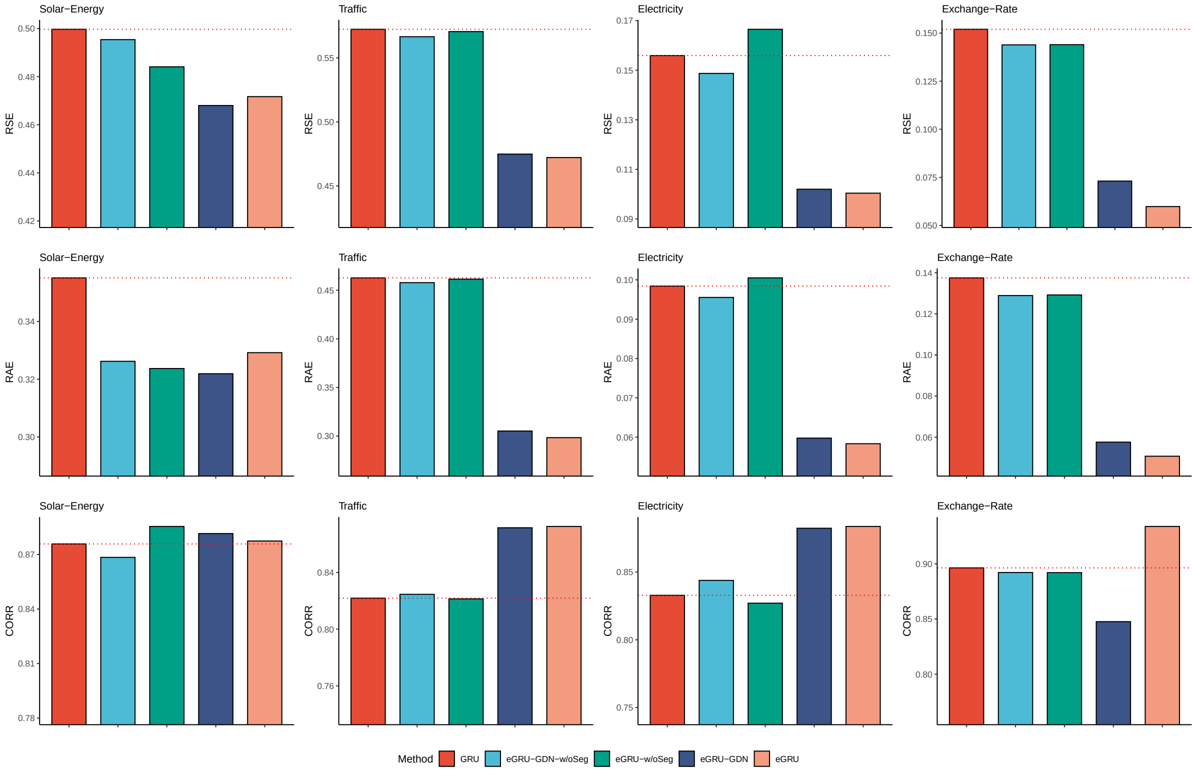

Figure S2. Ablation study of the components of eGRU: Extreme events labeling and Segment components on horizon 24. The mean values of GRU and eGRU are indicated by a dashed red and blue horizontal line, respectively.

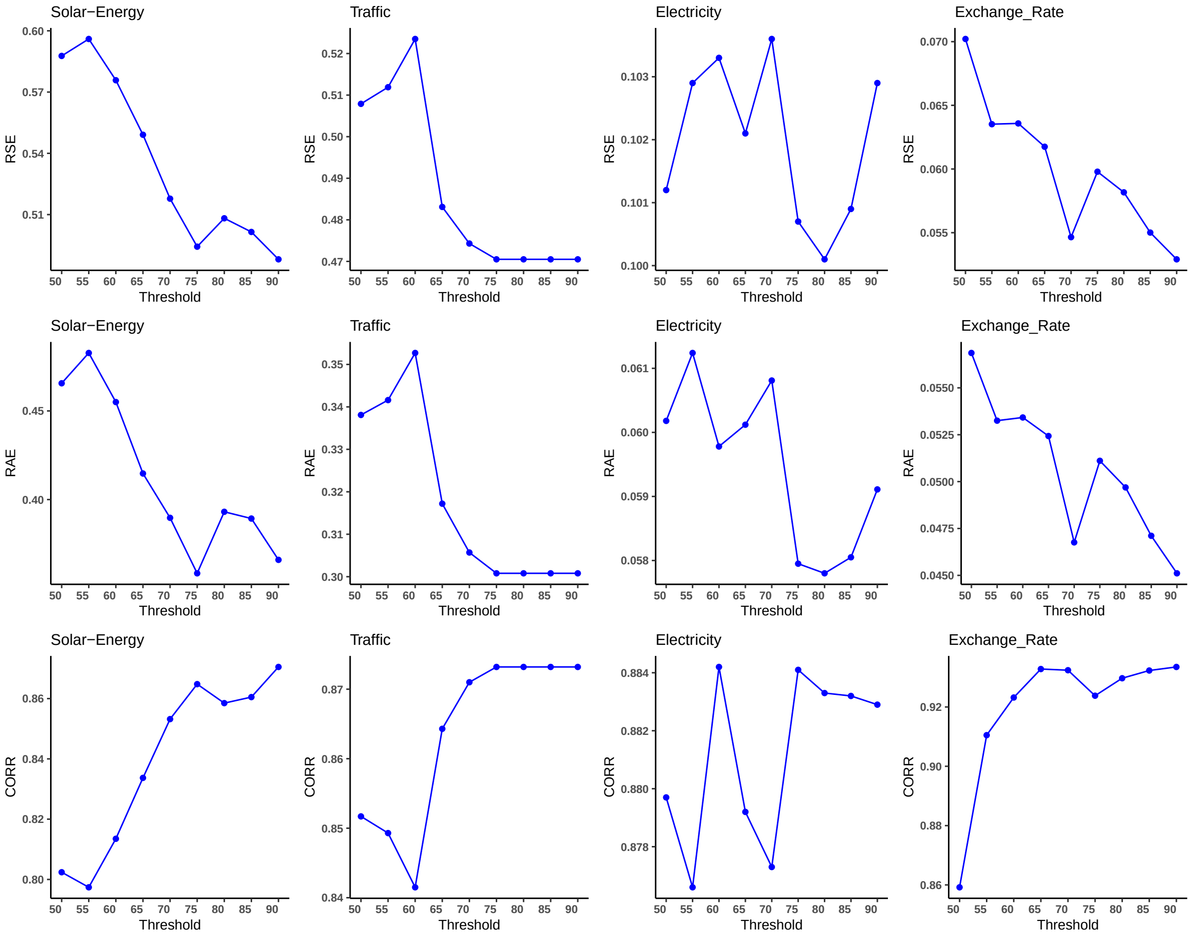

Figure S3. Evaluation on thresholds impact for forecasting accuracy on horizon 24. Please note that the scale of the y-axis is based on the range of values in the data.

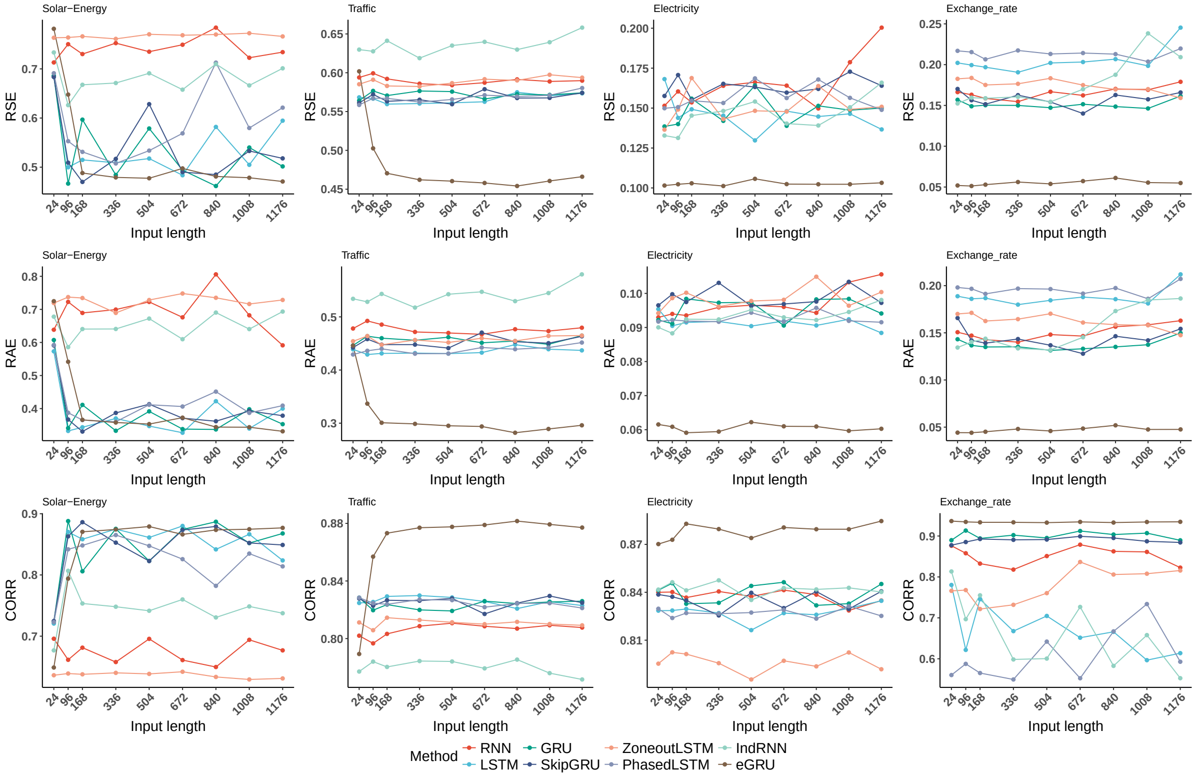

Figure S4. Evaluation on input sequence length impact for forecasting accuracy on horizon 24.

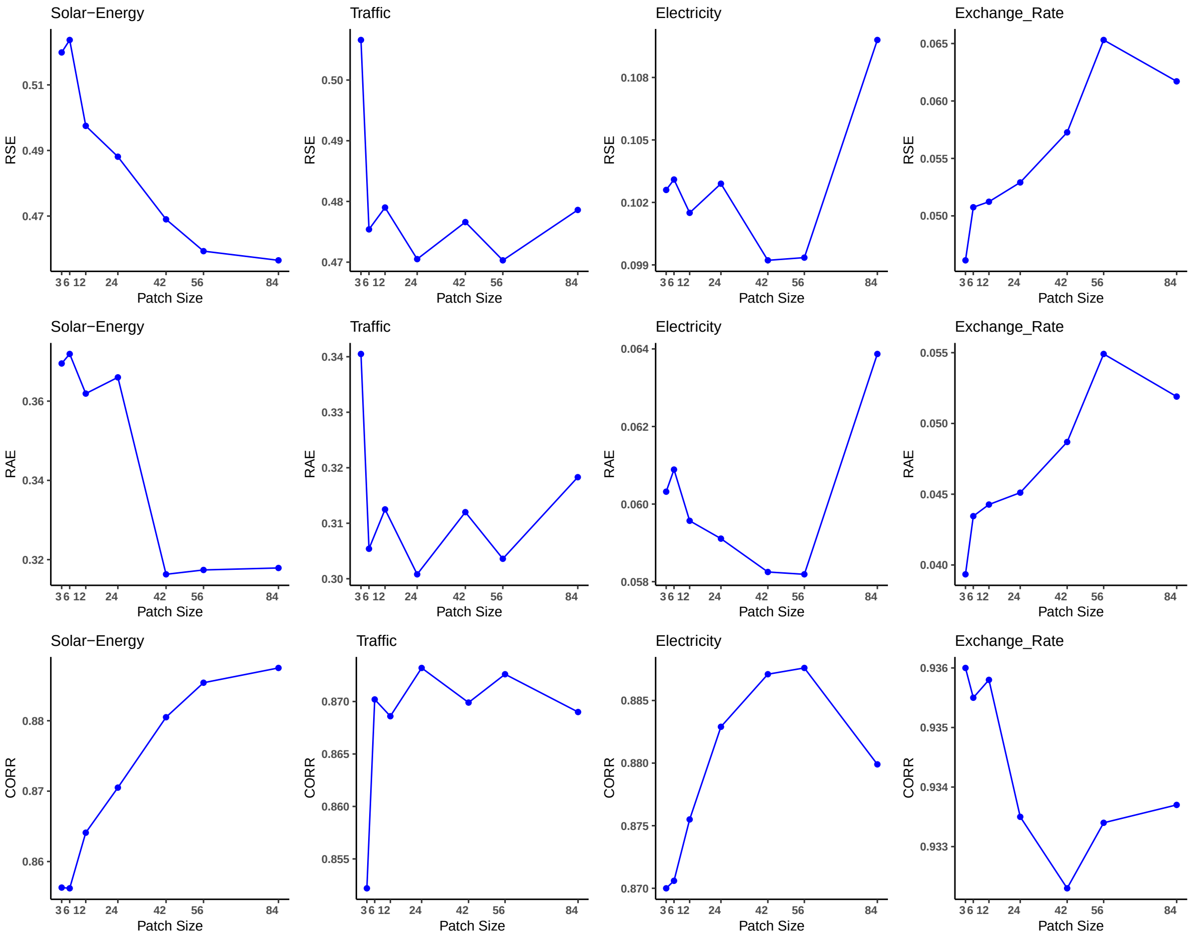

Figure S5. Evaluation on segmentation patch size impact for forecasting accuracy on horizon 24. Please note that the scale of the y-axis is based on the range of values in the data.

Method	Dataset	Solar-Energy		Traffic		Electricity		Exchange-Rate	
	Metric	MEAN	STD	MEAN	STD	MEAN	STD	MEAN	STD
RNN	RSE	0.7380	0.0084	0.5961	0.0058	0.1601	0.0075	0.1664	0.0093
	RAE	0.6978	0.0214	0.4903	0.0074	0.0950	0.0015	0.1513	0.0070
	CORR	0.6746	0.0110	0.8001	0.0040	0.8388	0.0023	0.8543	0.0276
LSTM	RSE	0.5410	0.0669	0.5618	0.0035	0.1489	0.0108	0.2072	0.0065
	RAE	0.3666	0.0518	0.4341	0.0029	0.0921	0.0021	0.1954	0.0070
	CORR	0.8529	0.0367	0.8279	0.0028	0.8263	0.0056	0.6323	0.0982
GRU	RSE	0.4852	0.0219	0.5700	**0.0026**	0.1575	0.0141	0.1570	0.0081
	RAE	0.3492	0.0220	0.457	0.0061	0.0959	0.0031	0.1435	0.0075
	CORR	0.8758	0.0138	0.823	0.0026	0.8362	0.0050	0.8517	0.0381
SkipGRU	RSE	0.5193	0.0240	0.5679	0.0080	0.1739	0.0177	0.153	0.0093
	RAE	0.3725	0.0196	0.4527	0.0104	0.1001	0.0037	0.1449	0.0066
	CORR	0.8588	0.0142	0.8240	0.0044	0.8341	0.0058	0.8680	0.0444
ZLSTM	RSE	0.7659	**0.0031**	0.5873	0.0029	0.1527	0.0164	0.1761	**0.0014**
	RAE	0.7261	0.0140	0.4545	**0.0027**	0.0988	0.0047	0.1654	0.0025
	CORR	0.6372	0.0036	0.8115	**0.0015**	0.7904	0.0162	0.7261	0.0353
PLSTM	RSE	0.5168	0.0362	0.5682	0.0081	0.1435	0.0118	0.2152	0.0112
	RAE	0.3700	0.0227	0.4397	0.0106	0.0910	0.0025	0.2004	0.0111
	CORR	0.8621	0.0160	0.8245	0.0050	0.8219	0.0049	0.6533	0.0793
IndRNN	RSE	0.6851	0.0175	0.656	0.0236	0.1540	0.0108	0.1526	0.0272
	RAE	0.6511	0.0263	0.5678	0.0352	0.0963	0.0050	0.1377	0.0262
	CORR	0.7454	0.0073	0.7735	0.0085	0.8452	0.0027	0.7794	0.0469
mLSTM	RSE	**0.4413**	0.0060	0.5524	0.0044	0.1488	0.0111	0.2246	0.0145
	RAE	**0.3047**	**0.0047**	0.4197	0.0037	0.0926	0.0021	0.2102	0.0137
	CORR	**0.8977**	**0.0018**	0.8346	0.0025	0.8205	0.0078	0.6438	0.1219
hsnLSTM	RSE	0.4726	0.0169	0.5264	0.0369	0.1308	0.0055	0.2570	0.0128
	RAE	0.3886	0.0194	0.5241	0.0309	0.0900	0.0009	0.2320	0.0105
	CORR	0.8806	0.0095	0.6493	0.0968	0.8093	0.0105	0.5832	0.0669
eGRU	RSE	0.4785	0.0054	**0.4728**	0.0051	**0.1006**	**0.0008**	**0.0582**	0.0024
	RAE	0.3457	0.0105	**0.3023**	0.0066	**0.0586**	**0.0007**	**0.0496**	**0.0018**
	CORR	0.8738	0.0028	**0.872**	0.0024	**0.8833**	**0.0008**	**0.9342**	**0.0003**

Table S1. Ablation study of random seeds

References

1. Deng, A. & Hooi, B. Graph neural network-based anomaly detection in multivariate time series. In *Proceedings of the AAAI Conference on Artificial Intelligence*, vol. 35, 4027–4035 (2021).

2. Siffer, A., Fouque, P.-A., Termier, A. & Largouet, C. Anomaly detection in streams with extreme value theory. In *Proceedings of the 23rd ACM SIGKDD International Conference on Knowledge Discovery and Data Mining*, 1067–1075 (2017).
